# Case Report: Early Identification of Subclinical Cardiac Tamponade in a Patient With a Left Ventricular Assist Device by the Use of Sublingual Microcirculatory Imaging: A New Diagnostic Imaging Tool?

**DOI:** 10.3389/fcvm.2022.818063

**Published:** 2022-03-25

**Authors:** Sakir Akin, Can Ince, Ard Struijs, Kadir Caliskan

**Affiliations:** ^1^Department of Cardiology, Unit Heart Failure, Heart Transplantation & Mechanical Circulatory Support, Erasmus Microcirculation (MC) University Medical Center Rotterdam, Rotterdam, Netherlands; ^2^Department of Intensive Care, Erasmus MC University Medical Center Rotterdam, Rotterdam, Netherlands

**Keywords:** left ventricular assist device, microcirculation, cardiac tamponade, heart failure, diagnosis

## Abstract

Clinical diagnosis of cardiac tamponade can be difficult in patients with continuous flow left ventricle assist devices (cf-LVADs). This is even more so because of the lack of adequate bedside echocardiographic windows. Previous studies on monitoring sublingual microcirculation showed deterioration of end-organ perfusion in patient with cardiogenic shock. In this paper we report alterations in the sublingual microcirculation in a cf-LVAD patient prior to clinical manifestation of tamponade. Our case report suggests that such real-time monitoring of the microcirculation may provide a new diagnostic modality for early recognition of cardiac tamponade.

## Introduction

The identification of (sub)acute cardiac tamponade following cardiac surgery is difficult to assess at the bedside. Patients may be (relatively) asymptomatic early in the course, but once intrapericardial pressure reaches a critical value limiting the cardiac output, the clinical course can be dramatic. In continuous flow left ventricle assist device (cf-LVAD) patients this is especially the case, due to the lack of pulsatility as a diagnostic indication. Recent studies observing sublingual microcirculatory alterations using incident dark-field (IDF) imaging may potentially provide a new bedside imaging modality for clinical assessment of shock (1, 2). IDF imaging consists of a hand-held device with a pen-like image guide probe (Braedius Medical, Huizen, the Netherlands) incorporating concentrically placed light emitting diodes incorporating IDF illumination with a set of high-resolution lenses projecting images on to a computer-controlled high-density image sensor synchronized to an illumination unit (3).

In this paper we report the presence of microcirculatory alterations in a cf-LVAD patient with cardiac tamponade before its clinical presentation.

## Case Description

**Figure 1:**
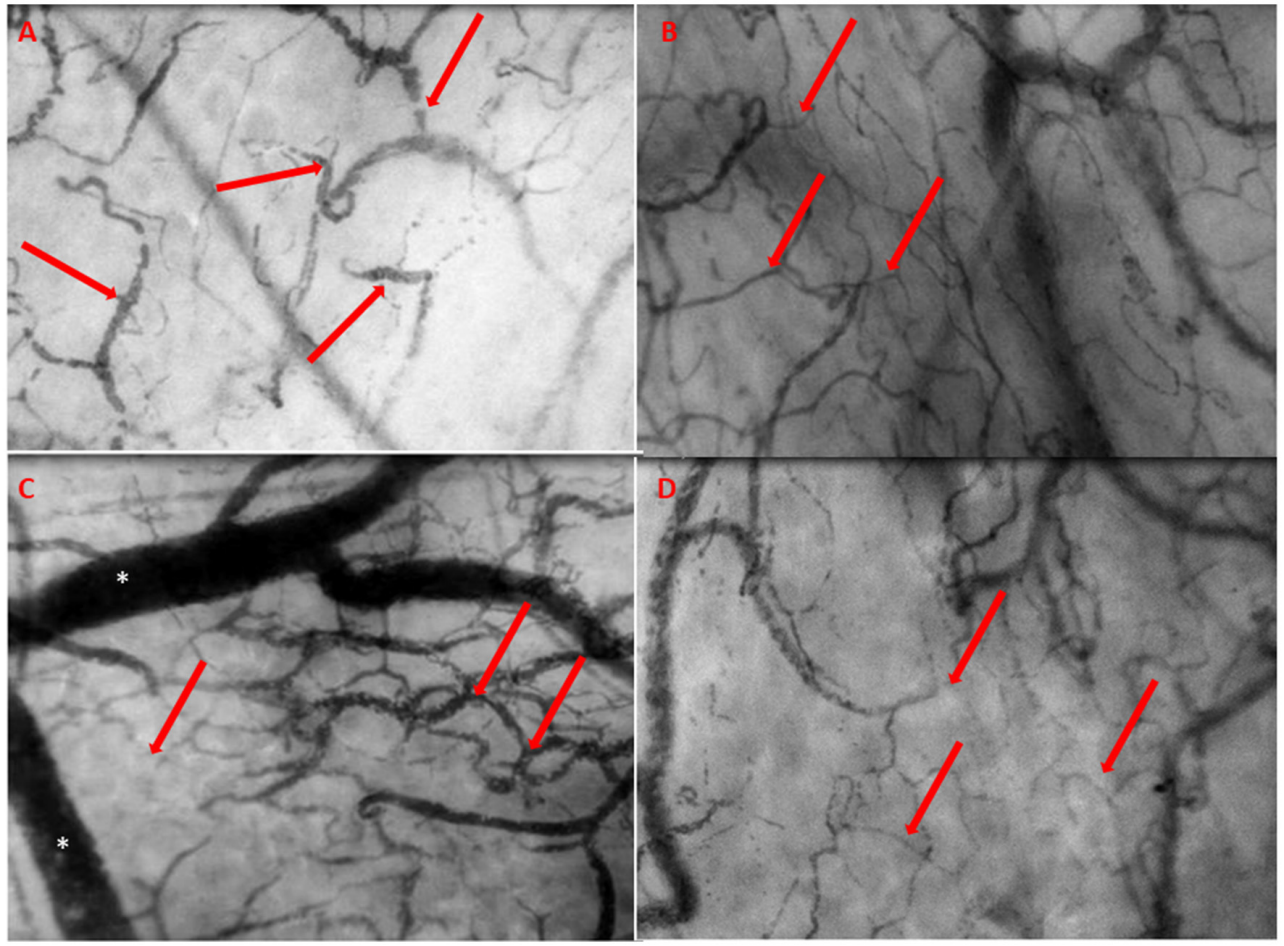
**(A)** The day prior to LVAD implantation. The microcirculation is typical as seen in heart failure characterized by slow, sludging flow (red arrows), stasis of red blood cells, and low capillary density. **(B)** The day after LVAD implantation with improved microcirculatory flow with a high red blood cell (RBC) velocity, concordant blood flow in all quadrants, and increased capillary density (red arrows). **(C)** The day of event, 10 days post-surgery, showing severe deterioration of microcirculation with severe stasis of red blood cells (red arrows) and severe congestion and distention of the venules (*). **(D)** Prior to discharge, quietly normalized microcirculatory flow (red arrows) after revealing cardiac tamponade.

**Figure 2:**
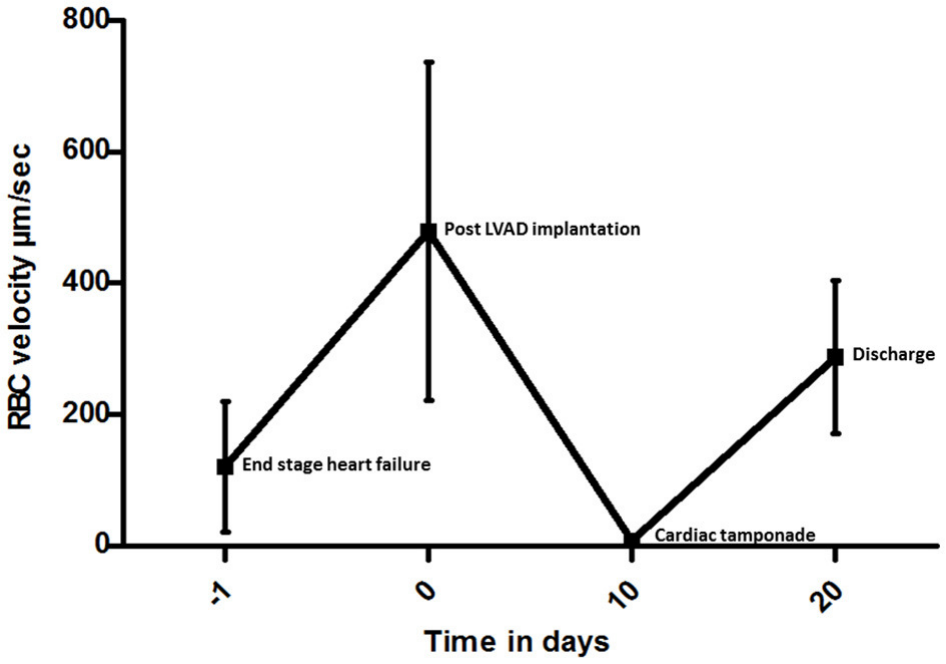
Space-time diagram analysis of red blood cell (RBC) velocity measured in five representative capillary video sequences at each time point in individual vessels from the day before LVAD to discharge. The RBC values are shown with bars giving the standard deviation.

A 39-year-old man, with a history of resection of the subvalvular aortic membrane and myectomy (30 years ago), was referred to our hospital because of progressive heart failure as a result of severe left ventricular (LV) systolic dysfunction with concomitant severe aortic regurgitation (AR). Conservative surgery with correction of AR was considered inappropriate due the severity of LV dysfunction. His exercise capacity appeared to be severely limited with a VO2 max of 13 kg/min (38% of predicted value). Due to further deterioration, a HeartMate II (Abbott, Chicago, USA) LVAD was implanted as a bridge-to-transplantation (BTT). His recovery was complicated by postoperative bleeding on day 5 requiring rethoracotomy. As part of a clinical research project, intermittent sublingually microcirculation measured using Cytocam-IDF imaging was initiated (4). The day before LVAD implantation, the sublingual microcirculation was typical as seen in the state of severe heart failure, characterized by slow, sludging movement of the red blood cells (Figure 1A and Supplementary Video 1). The day after LVAD implantation, the microcirculation improved significantly, with high red blood cell (RBC) velocity (Figure 1B and Supplementary Video 2). On day 10 after the LVAD implantation, the patient was apparently clinical stable with a heart rate of 90 beats/min, blood pressure of 99/73 mmHg, and normal diuresis. Also, his LVAD parameters were within the normal range: 8,600 rpm, flow 4.8 L/min (*N* = 3–10 L/min), pulsability index (PI) 7.0 ([(power max − power min)/power average] × 10), and power 4.7 Watt (*N* < 8 Watt). However, during this phase, microcirculatory imaging revealed a severe failure of microcirculatory function, reflected by a clear stasis of blood cells (Figure 1C and Supplementary Video 3). Later that evening the clinical condition of the patient deteriorated and clinical investigations were initiated. There was a situation with near collapse, followed by low flow alarms of the LVAD. The patient complained of dizziness and decreased PI of the HeartMate II. His blood pressure was 84/70 mmHg and his heart rate 90 beats/min. The transthoracic echocardiography showed pericardial thrombus formation at the posterior site without evident signs of tamponade. Given the unclear clinical status, an additional CT scan was performed showing evident signs of cardiac tamponade with thrombus formation around the right ventricle and posterior to LV. Subsequently a resternotomy was performed to relieve the cardiac tamponade. This surgical intervention resulted in a quick clinical recovery with restoration of microcirculatory blood flow as shown by sublingual measurements (Figure 1D and Supplementary Video 4). Additionally, off-line analysis of the microcirculatory movies using Automated Vascular Analyses software (AVA; MicroVision Medical^©^) allowed a quantitative analysis of the sublingual microcirculation and the velocity distributions to be made (5). Space-time diagram analysis showed that the velocity of red blood cell flow was significantly reduced during the event, and slowly recovered in the following days following treatment of cardiac tamponade until discharge (Figure 2). Further clinical recovery was uneventful.

## Discussion

(Sub)acute cardiac tamponade is a cardiac complication after open heart surgery, potentially lethal if diagnosed late (6). It usually results from accumulation of pericardial fluid, blood, and thrombus formation, leading to impaired cardiac filling and hemodynamic compromise. In this report, we present unique images of microcirculatory alterations in a cf-LVAD patient before the clinical manifestation of cardiac tamponade.

**Figure 3:**
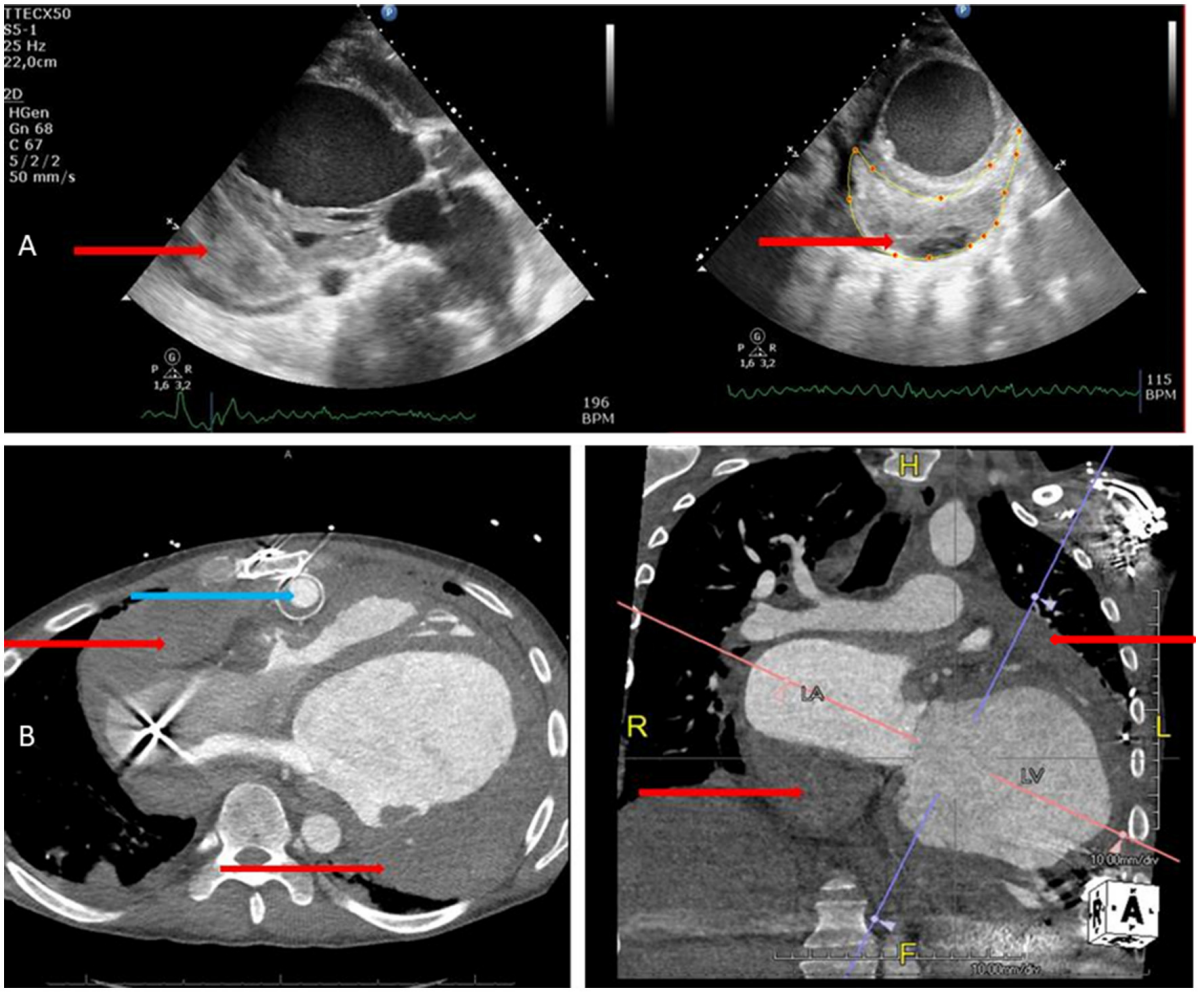
**(A)** Echocardiography images in parasternal long and short axis views showing thrombus formation (red arrows) in the pericardial space at the posterior area. **(B)** CT images of the whole thorax from transversal and anterior views showing thrombus formation (red arrows) around the left ventricle, and the atria images collected with echocardiography which are voluminous and more detailed and also show the HeartMate II outflow graft very clearly (blue arrow).

Postoperative bleeding and tamponade are considered major complications after implantation of cf-LVADs. Common hemodynamic characteristics of cardiac tamponade, including tachycardia, shock, or pulsus paradoxus, may be masked by the set values of the cf-LVAD (7). Transthoracic echocardiography is the first-line bedside cardiac imaging modality. However, due to blurred echo windows in LVAD patients, the diagnosis of pericardial effusion and resulting cardiac tamponade can be very challenging. However, echocardiography is readily available and portable, lacks ionizing radiation, and is highly sensitive for pericardial effusion. It is also very specific for diagnosis of pericardial tamponade in acute settings. If it does not result in feasible windows, computed tomography with additional information with assessment of the entire chest including associated abnormalities in the mediastinum, lungs, and adjacent structures is possible due to the larger field of view compared with that of echocardiography. IDF imaging is implemented in a hand-held device with a pen-like image guide (Braedius Medical, Huizen, the Netherlands) incorporating concentrically placed light emitting diode illumination when a set of high-resolution lenses projects images on to a computer-controlled high-density image sensor synchronized to an illumination unit (3). The patient-friendly and portable characteristics are comparable with that of echocardiography. Recently, IDF imaging has been validated for clinical assessment of microcirculatory alterations in critically ill patients (4). Further use of microcirculatory off-line analysis using Semi-Automated Vascular Analyses software (5) allows for quantitative analysis of sublingual microcirculation and the velocity distributions to be made (5). Using the method for space-time diagram analysis, significant alterations in the velocity of red blood cell flow were found. The images captured by echocardiography and a CT scan (8 h after sublingual microcirculatory alterations were present) after clinical deterioration during this cardiac tamponade are shown in Figure 3.

The study identified significant evidence for loss of hemodynamic coherence between the macrocirculation and microcirculation (8). Microcirculatory shock is the failure of microcirculation to support tissue perfusion and oxygenation, despite a normal restoration of systemic hemodynamics. A severely disrupted microcirculation might coexist with a restored systemic hemodynamic. Variables may not necessarily result in a correction of tissue and microcirculatory perfusion (9). Although this is to our knowledge the first description of microcirculatory IDF analysis in a patient with a continuous flow left ventricular assist device, there are limitations. This technique is not new and has to be implemented on larger populations with cf-LVADs. There are studies in the past that have described continuous flow circulatory support devices for temporary use (10–15). Furthermore, a limitation of our study is that we did not prove a direct causal effect between microcirculatory alterations and tamponade. Such alterations could also have been caused by acute right-sided heart failure, a pulmonary embolism causing severe obstructive shock, or in some cases, the condition of the pump itself, like pump thrombosis. Further technical limitations have to be taken into consideration as recently described (16). The assessment of red blood cell displacement as a measure of the microcirculatory convection capacity using current tools represents an even bigger challenge. Although measurement of the absolute red blood cell velocity of selected capillaries has been realized using manual space–time diagram analysis, applying this manual method to all capillaries rendered in an IDF image sequence for accurate and unbiased representation of red blood cell velocity within the field of view is not feasible (17).

In conclusion, in this case report we described the presence of microcirculatory alterations in a cf-LVAD patient in advance of the clinical manifestation of pericardial tamponade. Our study suggests that microcirculatory imaging may add a new modality in the arsenal of hemodynamic monitoring devices for identification of the early presence of tamponade in LVAD patients. This could probably add a new dimension in the early diagnosis and bedside monitoring of post-cardiac surgery patients, especially in patients with cf-LVADs.

## Patient Perspective

I am happy that doctors all over the world are learning from my case and I do not mind my condition being discussed. I do not want anyone to go through what I had to after an LVAD implantation.

As a young man with end-stage heart failure I have been given an LVAD implantation as a bridge to heart transplant. There were bleeding problems a couple of days after the operation in the ICU. There was a need for re-operation during my stay in the normal ward as well. Due to the LVAD in my thorax, echocardiography views were difficult to assess leading to CT scans. Dr. Akin asked me, as part of a clinical research project, to look under my tongue before the operation for intermittent microcirculation measurement using a camera. There were alterations seen in the microcirculation during my stay in the hospital before and after the LVAD implantation. For further optimizing the circulation after a LVAD implantation, such a technique could be useful in the future. Therefore, I give my consent for follow-up, during my out-patient clinical visits as well. It was very easy to measure this sublingually microcirculation by holding the camera with my hand and helping with the acquisition of the videos. I am very pleased with all the attention and care I received in the hospital. The doctors and nurses were nice to me. In the end, I hope that doctors all over the world have learnt something from reading my case.

## Data Availability Statement

The original contributions presented in the study are included in the article/Supplementary Material, further inquiries can be directed to the corresponding author/s.

## Ethics Statement

Ethical review and approval was not required for the study on human participants in accordance with the local legislation and institutional requirements. The patients/participants provided their written informed consent to participate in this study.

## Author Contributions

SA, CI, and KC performed the IDF imaging, contributed to the manuscript revision, participated in the design of the study, and contributed to the manuscript revision. AS participated in the interpretation of the data and drafted and revised the manuscript. CI was involved in the image analysis and the manuscript revision. All authors read and approved the final version of the manuscript.

## Conflict of Interest

CI has developed SDF imaging and is listed as an inventor on related patents that were commercialized by Micro Vision Medical (MVM) under a license from the Academic Medical Center (AMC). He receives no royalties or benefits from this license. He has been a consultant for MVM in the past but has not been involved with this company for more than 5 years and holds no shares or stock. Braedius Medical, which is a company that is owned by a relative of CI, has developed and designed a handheld microscope, namely, the CytoCam-IDF imaging microscope. The images used in the present study were obtained using this technology. CI has no financial relationship with Braedius Medical of any sort. Currently he is C.S.O. and holds shares in a company called Active Medical BV whose business is providing services and products related to clinical microcirculation. The remaining authors declare that the research was conducted in the absence of any commercial or financial relationships that could be construed as a potential conflict of interest.

## Publisher's Note

All claims expressed in this article are solely those of the authors and do not necessarily represent those of their affiliated organizations, or those of the publisher, the editors and the reviewers. Any product that may be evaluated in this article, or claim that may be made by its manufacturer, is not guaranteed or endorsed by the publisher.

## Supplementary Material

Supplementary Video 1 The day prior to LVAD implantation. The microcirculation is typical as seen in heart failure characterized by slow, sludging flow (red arrows), stasis of red blood cells, and low capillary density.

Supplementary Video 2 The day after LVAD implantation with improved microcirculatory flow with a high red blood cell (RBC) velocity, concordant blood flow in all quadrants, and increased capillary density (red arrows).

Supplementary Video 3 The day of event, 10 days post-surgery, showing severe deterioration of microcirculation with severe stasis of red blood cells (red arrows) and severe congestion and distention of the venules (^*^).

Supplementary Video 4 Prior to discharge, quietly normalized microcirculatory flow (red arrows) after revealing cardiac tamponade.
